# A Grounded Theory Model of Relationship Decision-Making in Non-Offending Partners of Individuals Accused of Sexual Offending

**DOI:** 10.1177/10790632231159075

**Published:** 2023-03-09

**Authors:** Lea C. Kamitz, Theresa A. Gannon

**Affiliations:** 1 Centre of Research and Education in Forensic Psychology (CORE-FP), School of Psychology, 2240 University of Kent, Canterbury, UK

**Keywords:** non-offending partners, sexual offending, grounded theory, romantic relationships, relationship dissolution

## Abstract

Non-offending partners of individuals who have committed sexual offenses often choose to end their relationship given the many negative consequences they face as a result of their partner’s offending behavior. Despite a focus on relationships in rehabilitation frameworks and the importance of the relationship for the individual who has offended and their partner, research has thus far failed to examine the process underlying why non-offending partners decide to stay in or leave their relationship following an offense. In this study we developed the first descriptive model of relationship decision-making in non-offending partners. Twenty-three individuals whose current or previous partners were accused of sexual offending were interviewed about affective, behavioral, cognitive, and contextual factors contributing to their decision to stay with or leave their partner. Participants’ narrative accounts were analyzed using Grounded Theory. Our resulting model consists of four main periods: (1) background factors, (2) relationship factors, (3) finding out, and (4) relationship decision-making. Clinical implications, limitations, and directions for future research are discussed.

Individuals who have sexually offended are seen by society as the worst of the worst (Quinn et al., 2004). Fueled by stereotypes perpetuated by the media (Malinen et al., 2013), the public displays extremely negative emotional reactions to the *sex offender* label and incorrectly assumes individuals who have sexually offended to be more resistant to treatment and more dangerous than individuals who have offended in other ways (e.g., Olver & Barlow, 2010; Rogers & Ferguson, 2011; Rothwell et al., 2021; Willis et al., 2010). Such stigmatization has been linked to negative outcomes, so-called collateral consequences (Burton et al., 1987), including loss of employment, housing issues (Tewksbury, 2005), depression (Brennan et al., 2018), self-harm, and suicide (Jeglic et al., 2013; Stinson & Gonsalves, 2014).

With raised awareness of their association with someone who has committed a sexual offense, non-offending family members may, through no fault of their own, experience what Goffmann (1963) termed *courtesy stigma*, a stigma by association (Condry, 2013). Consequently, society may treat non-offending family members and the individual who has offended “in some respect as one” (Goffmann, 1963, *p*.30). The courtesy stigma experienced by non-offending family members is a stigma by contamination combined with the stigma of their new identity as *family member of a sex offender* (Condry, 2013). Such stigma has been found to cause many collateral consequences for non-offending family members, similar to those experienced by those who have offended, leading family members to report feeling as if they themselves had been convicted of the offense (Farkas & Miller, 2007). These consequences include housing disruption, financial hardship, barriers to employment, social isolation, disruption of family bonds, and community harassment (Cassidy et al., 2021; Evans et al., 2021; Kilmer & Leon, 2017; Tewksbury & Levenson, 2009).

One of the groups most severely affected by courtesy stigma and its consequences is also one of the groups most commonly neglected by research: Non-offending partners of individuals who have sexually offended (Duncan et al., 2020). Stigma against non-offending partners often takes the form of blame as they are made responsible for omission, (i.e., not doing anything about an offense they were supposedly aware of; Liddell & Taylor, 2015), commission (i.e., not fulfilling their partner’s sexual needs and thus pushing them to offend), and continuation (i.e., not severing ties with their partner; Condry, 2013). As a result of stigmatization, non-offending partners experience similar practical, emotional, and social impacts to victims of crime (Brown, 2018; Duncan et al., 2020; Liddell & Taylor, 2015).

Practical consequences most commonly affecting non-offending partners are financial difficulties, employment issues, and residential impact, such as a lack of housing stability (Brown, 2018; Rapp, 2011), which may be exacerbated by media exposure (Duncan et al., 2020). Additionally, non-offending partners report significant psychological and emotional impact related to their partner’s offending behavior (Cahalane & Duff, 2018; Rapp, 2011). As most non-offending partners are unaware of the offenses, offense discovery can lead to trauma-, shock-, or bereavement-like responses (Duncan et al., 2022; Jones et al., 2021). Non-offending partners also report internalized stigma, self-blame, guilt, and shame, resulting from ongoing blame and stigma (Duncan et al., 2020, 2022). In the long-term, these factors have been shown to have a negative impact on non-offending partners’ mental health, leading to symptoms of depression and anxiety (Jones et al., 2021). There is also an immediate social impact on non-offending partners arising from their partner’s offending behavior and associated courtesy stigma. Non-offending partners report being harassed (Rapp, 2011) or ostracized by their community (Liddell & Taylor, 2015), and feeling like their family is being destroyed in cases where their identity is known (Cahalane & Duff, 2018). Others attempt to hide their stigmatized identity (Duncan et al., 2020) which leads to further isolation and a loss of social support (Jones et al., 2021; Rapp, 2011).

In many cases, isolation and stigma are exacerbated by intervening agencies, which non-offending partners perceive to be judgmental, insensitive, and ignorant (Duncan et al., 2020; Liddell & Taylor, 2015). For example, some support services appear to prioritize the “value” (Duff et al., 2017, *p*. 293) that non-offending partners hold in decreasing their partner’s recidivism risk (e.g., Duff et al., 2017; Shannon et al., 2013; Wager et al., 2015; see Hanson & Morton-Bourgon, 2005 and Larson et al., 2016 for research linking relationship breakdown and conflict to recidivism).

In most cases, the offense does not only immediately impact the non-offending partner but may cause a significant disruption to their relationship, as well. This may be triggered by the mourning of their partner’s psychosocial death (Bailey, 2018). While their partner is physically alive, the image the non-offending partner had of the person who has offended, and their idea of a future together, die (Duncan et al., 2020, 2022). While this evokes the same grief experienced by people who mourn a dead loved one, it is often not interpreted as valid by outsiders (Bailey, 2018). Additionally, non-offending partners report losing trust in their partner due to the offense (Cahalane & Duff, 2018). Often, outside influences can also lead to a disruption in the relationship. For example, Child Protection Services may prevent individuals who have sexually offended from residing in the same household as the non-offending partner (Duncan et al., 2020).

Given these relationship problems, the cognitive effort required to maintain a positive view of the relationship (Duncan et al., 2022), and the courtesy stigma they experience, it is unsurprising that non-offending partners may choose to end their relationship. While there is a lack of literature to decisively conclude how many non-offending partners end their relationships, qualitative explorations have suggested that less than 50% of those who have sexually offended may remain in their relationship post-conviction (Lytle et al., 2017). Relationship breakdown is more common for individuals who have committed sexual offenses than those who have committed non-sexual offenses, especially if the victim was a child (Farmer et al., 2015; McAlinden et al., 2017). For non-offending partners, who, unlike other family members of those who have offended, are not considered genetically contaminated (Condry, 2013), leaving the relationship is the only way to end courtesy stigma (Jones & Giles, 2022). Additionally, professionals and the community often urge non-offending partners to leave their relationship (Duncan et al., 2020; Jones & Giles, 2022) and ostracize those who fail to do so (Jones & Giles, 2022). Yet non-offending partners may be unable to make relationship decisions immediately due to trauma, shock, and confusion (Duncan et al., 2020).

Considering the importance of the relationship for both the person who has offended and the non-offending partner, it is surprising that so far, research has failed to thoroughly examine the process underlying why non-offending partners decide to stay with or leave their partner (Iffland et al., 2016). Given the dearth of research in this field, the main aim of this study was to develop the first descriptive model of the decision-making process underlying relationship termination or continuation in non-offending partners of individuals who have been accused of sexual offending. Grounded Theory was considered ideal as a method for developing such a model since it may be used for inductive analysis of relatively small amounts of qualitative data in areas where existing theory and research is sparse (Glaser & Strauss, 1967; Ward et al., 2006).

Thus, using Grounded Theory, we aimed to describe the contributions of affective, behavioral, contextual, and cognitive factors to the decision to stay in or leave a relationship after finding out that a partner has been accused of a sexual offense. We expected that such a theory would contribute to academic literature by addressing the current lack of research in this field (Duncan et al., 2020; Iffland et al., 2016) and inform professionals working with non-offending partners about their unique situation and needs. It may also inform rehabilitation frameworks for individuals who have sexually offended (e.g., Risk-Need-Responsivity Model, Bonta & Andrews, 2017; the Good Lives Model, Ward & Gannon, 2006) which place emphasis on relationships.

## Method

### Ethics

Ethical approval was obtained from the University of Kent School of Psychology Ethics Committee. Prior to the interview, informed consent was given by each participant via a signed consent form. Following the interview, participants were provided with details of community support groups and organizations relevant to their respective location and given a full debriefing.

### Participant Recruitment

We recruited both those whose partners were charged with or convicted of sexual offending, and those whose partners were accused. The reasons for this were twofold: First, sexual offenses are underreported (U.S. Department of Justice, Office of Justice Programs, Bureau of Justice Statistics, 2016), and even those that are investigated by the police often do not result in any charges or a conviction (Crown Prosecution Service, 2022). Second, individuals who have sexually offended commonly minimise parts of their offending (Schneider & Wright, 2004; Ward et al., 1995), or categorically deny that they have offended, especially in cases where the victim was a child (Ware et al., 2020). Previous studies with small samples of non-offending partners have shown that they may exhibit similar cognitive distortions (Cahalane & Duff, 2018), particularly when choosing to remain in the relationship (Duncan et al., 2022). We did not want to exclude such potential participants to provide a balanced account of the experiences of non-offending partners, especially as a belief in the accusations or a denial of them may be a factor in the relationship decision-making process.

Five participants were recruited through leaflets distributed in different UK city centers and universities, and another six participants approached the researchers via their social media accounts or heard about the study by word of mouth. They completed a pre-screen through Qualtrics and arranged a telephone interview. The remaining 12 participants were recruited through the crowdsourcing platform Prolific. Those participants had completed the pre-screen, which was open to all Prolific users, for a reimbursement of £0.12. We made appointments for Zoom audio- or video-calls with eligible participants through Prolific’s anonymous messaging system. Upon interview completion, all participants, regardless of recruitment method, received a reimbursement of £20, either through Prolific, or as an Amazon voucher.

### Participants

We recruited 23 participants (20 females and three males), with ages ranging from 19 to 52 years (*M =* 31.87, *SD =* 11.00). Most participants were white (British, Irish, North American, or “Other”; 79.26%, *n =* 18), two were Asian (Indian or “Other”, 8.7%), two were Middle Eastern (8.7%), and one was Black (African, 4.35%). At the time they found out about the accusations, most participants (*n* = 16, 69.57%) resided in the United Kingdom.

#### Relationship Characteristics

Twenty-one of the participants’ accused partners were male, and two were female. Prior to finding out about the accusations, the duration of the participants’ relationships ranged between one month and 240 months or 20 years (*M* = 57.65 months, *SD* = 76.22 months, *Mdn = 12 months*). The participants’ total relationship duration ranged from 5 months to 264 months or 22 years (*M* = 73.96 months, *SD* = 80.37 months, *Mdn* = 36 months). Fourteen participants were in self-defined long-term relationships and the remaining nine participants were married to their partner when they found out about the accusations. Six participants had at least one child that they were raising with their partner, whereas 17 participants did not have children with their partner who was accused.

Seven participants immediately decided to end the relationship after learning about the accusations. Of those, four participants later reconciled with their partner. The remaining 16 participants decided to stay in the relationship after finding out about the accusations. At the time of interview, however, most participants (*n* = 17) were not in the relationship anymore.

#### Characteristics of the Accusations

All participants reported that they were in the relationship when they first learned of the sexual offense accusations against their partner. However, the alleged offenses were said to have taken place prior to the relationship for seven participants. For two participants, their partners’ alleged offenses were said to have taken place both during and before the relationship. For the remaining 14 participants, the accusations pertained to offenses that were alleged to have occurred during the relationship.

The majority (*n* = 20) of participants’ partners were accused of one offense, while a few (*n* = 4) were accused of committing multiple offenses. We classified these offense accusations in line with UK legislation. Fourteen partners were accused of contact offenses against adults, of which nine were accused of sexual assault (excl. rape), and six were accused of rape (Sexual Offences Act, 2003; §§ 3 & 1). Two partners were accused of committing non-contact, internet-based offenses against adults. Here, both were accused of disclosing private sexual photographs or films with intent to cause distress (Criminal Justice and Courts Act, 2015; § 33). One participants’ partner was accused of committing contact offenses against children, both rape and sexual assault of children under 13 (Sexual Offences Act, 2003; §§ 5 & 7). Lastly, six partners were accused of non-contact, internet-based offenses against children. Here, four participants’ partners were accused of sexual communication with a child, one was accused of attempted meeting a child following sexual grooming (Sexual Offences Act, 2003; §§ 15A & 15), and four were accused of possession of indecent photographs of children (Protection of Children Act, 1978; § 1). None of the accusations against participants’ partners pertained offenses against an intrafamilial victim.

Eight participants believed the accusations against their partner to be true when they first learnt of them. Of the 11 participants who did not believe them to be true initially, nine believed the accusations to be true by the time of interview.

The accusations against 11 participants’ partners were not formally investigated by the responsible Criminal Justice System, although two of these led to consequences for the participant’s partner as drawn by an authoritative body that was not related to the Criminal Justice System (e.g., being banned from a university campus). In the remaining 11 cases, a Criminal Justice System investigation led to no charges for two participant’s partners. One participant’s partner was found ‘not guilty’ during court proceedings, one received a probation order, one received a suspended sentence, and six received custodial sentences.

For a summary of relevant information about each participant, see Table 1.

**Table 1.** Participant Information.

Participant	Age	Country of Residence	Accusations Against Partner	Victim Age	Criminal Justice Response	Relationship Decision	Current Relationship
P1	19	United Kingdom	Contact	Adult	None	Left, later reconciled	Separated
P2	19	United Kingdom	Contact	Adult	None	Stayed	Separated
P3	23	United Kingdom	Contact	Adult	Custodial sentence	Left	Separated
P4	21	United Kingdom	Contact	Adult	None	Stayed	In relationship
P5	20	United Kingdom	Contact	Adult	None	Left, later reconciled	In relationship
P6	26	United States	Contact	Adult	None	Stayed	Separated
P7	42	Ireland	Contact	Child	Custodial sentence	Left	Divorced
P8	31	United Kingdom	Contact	Adult	None	Stayed	Widowed
P9	29	Israel	Contact	Adult	None	Stayed	Separated
P10	42	United Kingdom	Contact	Adult	Investigated, but no charge	Stayed	Divorced
P11	28	Greece	Contact	Adult	None	Stayed	Separated
P12	23	Ireland	Non-contact (Internet)	Adult	None	Stayed	Separated
P13	48	South Africa	Contact	Adult	Investigated, but no charge	Stayed	Separated
P14	29	United Kingdom	Contact	Adult	Partner found ‘not guilty’	Stayed	Separated
P15	23	United Kingdom	Non-contact (Internet)	Adult	Custodial sentence	Left	Separated
P16	34	United Kingdom	Contact	Child	Probation order	Stayed	Divorced
P17	25	United Kingdom	Contact	Adult	None	Stayed	Separated
P18	21	United States	Contact	Adult	None	Stayed	Separated
P19	45	United Kingdom	Non-contact (Internet)	Child	Custodial sentence	Stayed	Separated
P20	52	United Kingdom	Non-contact (Internet)	Child	Suspended sentence	Stayed	Married
P21	41	United Kingdom	Non-contact (Internet)	Child	Custodial sentence	Left, later reconciled	Married
P22	40	United Kingdom	Non-contact (Internet)	Child	Custodial sentence	Left, later reconciled	Married
P23	52	United Kingdom	Non-contact (Internet)	Child	Suspended sentence	Stayed	Married

*Note.* Country of Residence = country the participant resided in when finding out about the accusations. Current Relationship = relationship status with participant’s (ex-) partner at the time of interview.

### Data Collection

All interviews were conducted by the first author. During the interview, participants were first asked to detail potentially important aspects of their childhood and adult life (e.g., major life events, family background, peer- and intimate relationships). Afterwards, participants were asked to describe events leading up to, during, and following the knowledge of the accusation(s) against their partner. The focus was on the participants’ thoughts and feelings, and on factors which they expressed to be important in their decision to stay with or leave their partner. A semi-structured interview schedule was used as a guideline to ensure important aspects of the participant’s narrative were covered while allowing the participants to lead the interview and expand on issues they deemed important. Due to this participant-led approach, and the uniqueness of each individual narrative, the length of the interviews varied from 41 to 127 minutes (*M* interview time = 74:07, *SD* = 21:40). Interviews were recorded with the participants’ knowledge and consent. All interviews were subsequently transcribed verbatim by the first author.

### Analysis

First, each transcript was divided, line by line, into its most basic units of meaning, which are commonly referred to as *meaning units* (Strauss & Corbin, 1998). See below an example of how an interview extract was broken down into several meaning units (illustrated using a slash):

“But I was really shocked.^1^/ And I was just apologizing, like it was my fault.^2^/ Like, I felt responsible for some reason, even though I never met her or anything.^3^”

Subsequently, during *open coding* (Strauss & Corbin, 1998), raw data was broken down into their conceptual components and meaning units were abstracted into more general meaning units, by allocating descriptive labels to each meaning unit. This was done primarily by coding in gerunds which, in contrast to coding in topics and themes, enables the researcher to study processes, actions, and implicit connections (Charmaz, 2014). Thus, meaning unit ^3^ from the sample above became “feeling responsible despite never having met her.”

Every general meaning unit was then assigned one or more low-level concepts, which represent the ideas contained in the data. Subsequently, during *axial* coding, these concepts were linked with each other and arranged into categories, higher-level concepts, based on conceptual similarity and shared characteristics. Thus, meaning unit^3^ was assigned the low-level concept “feeling guilty”, and the category “reaction to accusations”. Since Grounded Theory is cyclical in nature, during *comparative analysis* new concepts and categories were generated and refined as they were encountered throughout the analytical process. Following this, the relationships between major categories were identified. Along with their respective concepts, categories were ordered chronologically and integrated into a preliminary model illustrating events before, during, and after an individual’s decision to stay with or leave their partner (*theoretical integration)*. This process was followed for the first 16 transcripts. The remaining seven transcripts were used to cross-validate our preliminary model by comparing each transcript’s fit with the already developed categories of the model. During this process of *constant comparison,* existing categories were further refined, and new categories were added where necessary. Validation also ensured that *saturation* was reached, that is, no further data emerged from new transcripts and the model was fully developed, reflecting the relationship decision-making process in our sample.

To minimize any potential biases arising from prior assumptions about the topic, the first author, who had no previous work experience or contact with non-offending partners or individuals who have sexually offended, completed all the data collection and initial meaning unit analysis. Throughout data collection, analysis, and write-up of the findings, the authors additionally engaged in reflexivity by reflecting on and challenging their potential biases and emotions towards the topic.

## Findings

Our final model chronologically describes the feelings, thoughts, behaviors, and contextual events leading up to and following our participants’ decision to stay with or leave their partner after finding out that their partner had been accused of a sexual offense. As such, the model can be broken down into five periods: (1) *Background Factors;* the participant’s childhood, adolescence, and adulthood experiences up until the point they met their partner who was accused of a sexual offense, (2) *Relationship Factors;* factors associated with the participant’s relationship prior to them finding out about the accusations, (3) *Finding Out;* factors that occur immediately before, during, and after finding out about the accusations, and (4) *Relationship Decision-Making*; factors associated with whether the participant decided to stay with or leave their partner.

### Period 1: Background Factors (see Figure 1)

**Figure 1.**
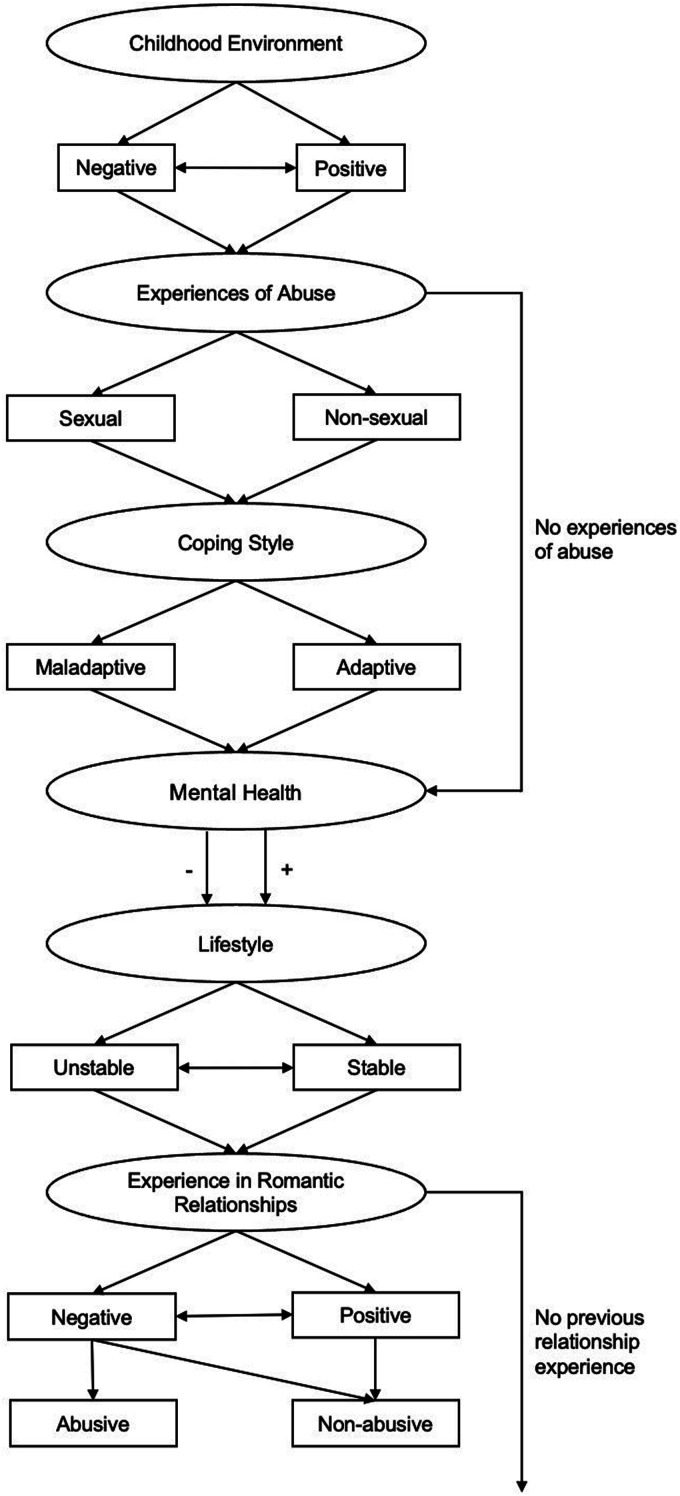
Period 1: Background factors.

The data which emerged regarding participants’ backgrounds was divided into six main categories with additional subcategories (see Figure 1). The first category to emerge was *Childhood Environment*. This encompasses early childhood experiences including relationships with caregivers and peers. A participant’s childhood environment was either mostly negative (*n =* 9; e.g., experiences of trauma, poor caregiver relationships, absent caregivers, poor parental mental health, poverty) or mostly positive (*n* = 7; e.g., stable and loving caregiver relationships, positive relationships with peers). Some participants experienced a considerable improvement (e.g., being taken care of by grandparents after suffering neglect by parents) or deterioration (e.g., deteriorating relationship with caregivers, parental death, sudden financial instability) of their childhood environment (*n* = 7). This is depicted by the double-ended arrow connecting the negative and positive *Childhood Environment* pathways in Figure 1. Those who had a negative childhood environment, and especially those who lacked support from their caregivers in childhood, would often come to rely on romantic partners for support in later life, whereas those who had a positive childhood environment tended to receive support from their wider family.

For those who described a negative childhood environment at any point, including those who experienced a considerable improvement or deterioration of their childhood environment, *Experiences of Abuse* at some point during their childhood and adolescence were also common (*n* = 15). These were either sexual (*n* = 5; e.g., sexual abuse through peers or adults, often repeated victimization) or non-sexual (*n* = 10; e.g., intense bullying, neglect, physical abuse by family members, witnessing domestic abuse) in nature. For many participants who had experienced abuse, this wove its way throughout their lives in the form of repeated victimization by romantic partners, family, and peers. Eight participants reported not experiencing any abuse during their childhood as depicted by the arrow to the right of *Experiences of Abuse,* feeding into the fourth main category of *Mental Health*. Participants who did not experience abuse reported feeling cared for and respected by their peers and family.

*Coping Style*, the third main category in this period, describes how those participants who were abused in childhood coped with such experiences. While many participants reportedly developed maladaptive coping styles (*n* = 10; e.g., repressing memories of abuse, minimizing abuse, using substances), some reported adaptive coping mechanisms (*n* = 5; e.g., therapeutic intervention, exercise, leaving an abusive environment).

The fourth main category to emerge in this period was *Mental Health.* Participants described having had either predominantly negative or positive *Mental Health* during their childhood and adolescence. Most participants (*n* = 14) reported some poor *Mental Health* as characterized, for instance, by depression, anxiety, eating disorders, and personality disorders. The remaining nine participants described having good *Mental Health* (i.e., lack of psychiatric diagnoses, resilience to challenging life events). Those participants who experienced a negative or mixed *Childhood Environment* and had *Experiences of Abuse* were more likely to experience poor *Mental Health* than other participants.

For some participants, this translated into an unstable *Lifestyle* (*n* = 5) associated with dropping out of school, abusing substances, being homeless, unwanted pregnancy, and not having a stable place of work or social support. Most participants (*n* = 4) who experienced an unstable *Lifestyle* were able to stabilize their *Lifestyle* before entering the relationship with their partner who was accused of a sexual offense (e.g., by finding work after dropping out of school). This is depicted by the arrow connecting the stable and unstable *Lifestyle* pathways in Figure 1. Most participants (*n* = 18) described having a stable *Lifestyle* including academic achievement, stable peer relationships, and stable employment.

The final main category to emerge in this period was *Experience in Romantic Relationships.* This encompasses any experiences in romantic relationship participants had before meeting their partner who was accused of a sexual offense. Most participants had *Experience in Romantic Relationships (n* = 19) and reported this to be either predominantly negative (*n* = 11) or positive (*n* = 3). Negative *Experience in Romantic Relationships* was often characterized by instability, unhappiness, and feeling like one’s needs were not met (e.g., “I feel like I’ve had experience beyond my years, as they say, in the ‘unlucky’ and ‘with men’ department”). Predominantly positive *Experience in Romantic Relationships* was much rarer and usually consisted of stable, consensual, and fulfilling romantic relationships (e.g., “It was the first time that I understood that I want to continue to feel those feelings for the rest of my life”). Some participants (*n* = 5) had both positive and negative *Experience in Romantic Relationships,* as depicted by the arrow connecting the negative and positive *Experience in Romantic Relationship* pathways in Figure 1. For instance, some participants described “sabotaging” their relationships, or having relationships which were initially positive turn negative (e.g., “He was caring, he was handsome. Until, I guess, he got tired of me and started cheating.”). Four participants reported not having had any previous relationship experiences as depicted by the arrow to the right-hand side of *Experience in Romantic Relationships.* For these participants, the relationship with their partner who was accused of a sexual offense was their first romantic or sexual relationship. Participants who had no previous *Experience in Romantic Relationships* reported being naïve about relationships and not knowing what would be considered “normal” and healthy in a relationship (e.g., “It was my first relationship, I don’t really have anything to compare it to. So, it was a lot of asking my friends and my sister ‘Is this good? Do you like him? Is he nice?’”).

Some participants (*n* = 5) who reported having had a negative *Experience in Previous Relationships* also described these relationships as abusive. This abuse could be sexual (e.g., “All they want to do is try and pressurize you into having sex”), emotional (e.g., “It never felt predatory, but looking back at it, there was definitely a strange power dynamic that he was exploiting”), or physical (e.g., “He had split my head open at the back”), and some participants were repeatedly victimized by multiple partners. For some participants, this influenced how they evaluated subsequent relationships, especially the relationship with their partner who was accused of a sexual offense (see Period 2). However, most participants who had *Experience in Previous Relationships* (*n* = 14) did not report any abuse occurring in these relationships.

### Period 2: Relationship Factors (see Figure 2)

**Figure 2.**
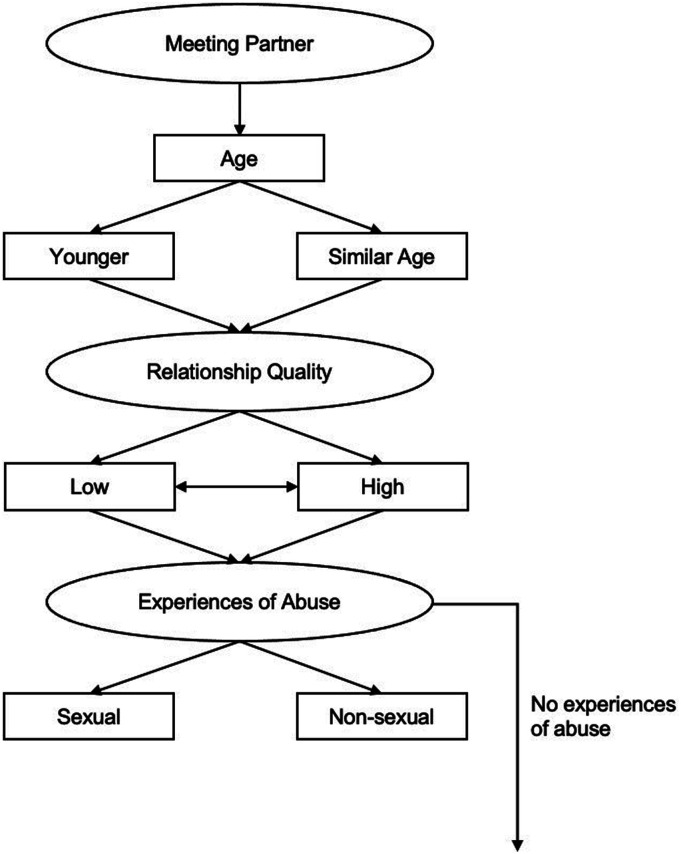
Period 2: Relationship factors.

The data which emerged regarding the participants’ relationship with their partner who had been accused of a sexual offense was split into four main categories, with additional related concepts for some of these categories (see Figure 2). The first category to emerge, *Meeting Partner,* encompasses factors related to when and how the participant met their partner who would later be accused of a sexual offense. Participants reported that their *Age* at meeting, when compared to their partner, played a significant role in their relationship dynamic. Participants who were significantly younger than their partners (*n* = 7) often had less experience in relationships and reported their partner assuming an almost parental role towards them. This power imbalance was at times used by the participants’ partners to abuse and manipulate them (e.g., “I felt like he almost became a parent. If I did something that he thought was wrong, then I would be verbally reprimanded.”). In comparison, participants who were of a similar *Age* to their partner (*n* = 16) reported being in a similar stage of their lives as their partner and subsequently feeling more equal to their partner.

The second category to emerge regarding participants’ relationship factors was *Relationship Quality*. The quality of the relationship was either described to be predominantly low (*n* = 6; e.g., unhappiness, high levels of conflict, distant partner) or high (*n* = 9; e.g., stable relationship, contentment, happiness, seeing the partner as a “good person”, resolving conflicts constructively). For many participants (*n* = 8) *Relationship Quality* fluctuated throughout the relationship, as depicted by the double-ended arrow connecting low and high *Relationship Quality.* Participants were generally more likely to leave the relationship following the accusations if they perceived the *Relationship Quality* to be low. Some reported that they viewed the accusations as a “way out” of an already unhappy relationship. However, an exception to this were cases in which the relationship between the participant and their partner was abusive.

*Experiences of Abuse* were either sexual (*n* = 2; e.g., assault, rape) or non-sexual (*n* = 5; e.g., emotional abuse, physical abuse, manipulation). Participants who were abused by their partner reported, later down the model, being manipulated into believing their partner over any evidence they might have seen and consequently denying their partner’s offense. They appeared to also fear the consequences of leaving their partner. Most participants (*n* = 16), however, did not describe any *Experiences of Abuse*, as depicted by the arrow on the right-hand side of the model. This led participants who had experienced abuse in prior relationships (see Period 1) to evaluate the relationship with their partner who was accused of a sexual offense to be especially positive, since they reported feeling lucky to have finally found a non-abusive partner (e.g., “There was no abuse. There was, you know, I felt, Jesus Christ, I’ve hit lucky here”, “So, he was different from the lads. This guy was very easy-going, wasn’t telling me what to do.”).

### Period 3: Finding out (see Figure 3)

**Figure 3.**
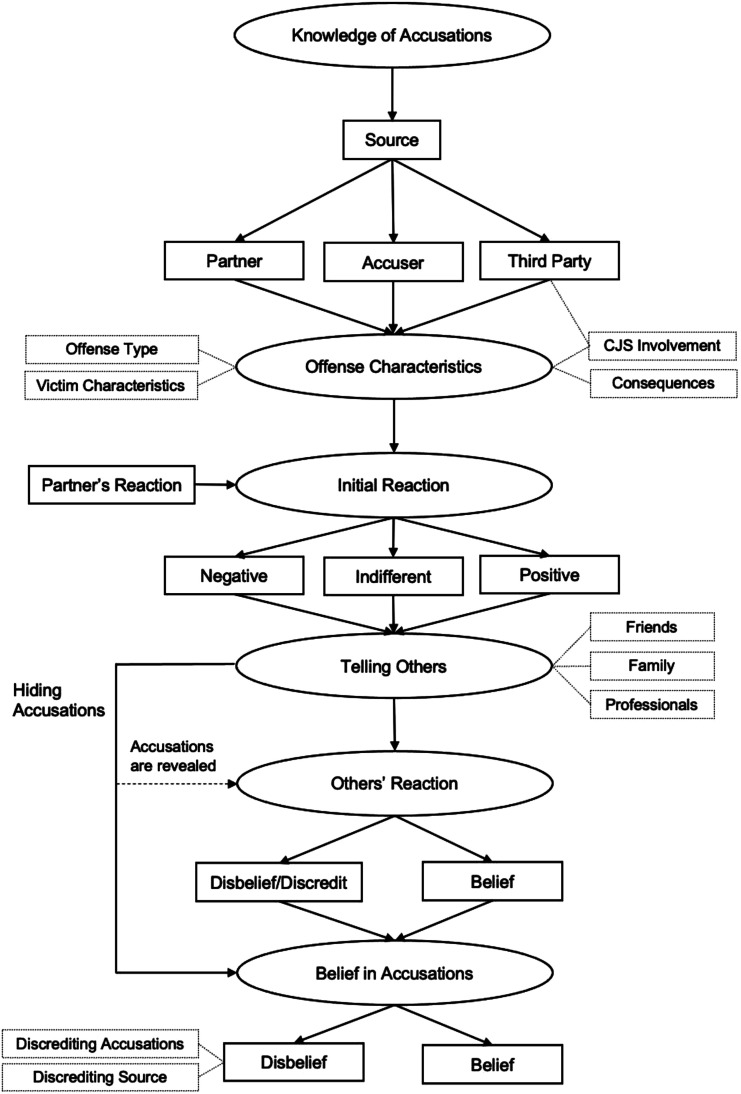
Period 3: Finding out.

The data which emerged regarding the participants finding out about the accusations against their partner was divided into six main categories, with further related concepts (see Figure 3). The first category which emerged was the *Knowledge of Accusations,* which encompasses the factors related to the circumstances surrounding the participants finding out about the accusations. The main concept found to be of importance within this category was the *Source* of the accusations. This was either the participant’s partner themselves (*n* = 5), the accuser (*n* = 7), or a third party (*n* = 12). If the participant’s partner confessed to having committed an offense, the participants were more likely to believe the accusations. However, the participant’s partner may also warn the participant of what they claim to be false accusations, which made participants less likely to believe the accusations. The *Source* could also be the accuser. When this was the case, the accuser often reached out to the participant to warn them about their partner and what they may be capable of. Whether the participant believed the accuser highly depended on the accuser’s characteristics, as outlined in the concept “*Victim Characteristics*”, under the *Offense Characteristics* category. Lastly, the participant may also have been made aware of the accusations by a third party, who was not directly involved in the incident. In most cases, this was either a witness, or an involved authority, namely the Criminal Justice System (*n* = 6). While some participants described such Criminal Justice System involvement as traumatic, others had more positive, and supportive experiences. The engagement of the Criminal Justice System also appeared to affect whether the participant believed the accusations. For example, if the police portrayed the accusations to be lacking credibility, or a jury found their partner “not guilty”, participants were more likely to believe that the accusations were false.

The second major category to emerge was *Offense Characteristics*. When considering *Offense Type*, contact offenses were often viewed as more serious than non-contact offenses, or online offenses. Another important concept was *Victim Characteristics* (e.g., victim age, mental illness, victim – perpetrator relationship, victim – participant relationship). The younger the accuser or victim was at the time of the alleged offense, the more likely the participant was to take such accusations seriously. Additionally, if the accuser had previously been in a relationship with the participant’s partner, they were more likely to be discredited and disbelieved. This was due to the assumption that they made the accusations to “punish” the participant’s partner for a breakup or wanted to break up the participant’s relationship to win back their former partner. As previously mentioned, *Consequences*, such as those arising from Criminal Justice System involvement, could also heavily impact a participant’s perception of accusations. Such Criminal Justice System involvement (e.g., imprisonment) appeared to make the accusations more credible. No formal consequences or even acquittal seemingly had the opposite effect, with participants reporting that they believed the Criminal Justice System to make the “right” decision (e.g., “A jury of people have listened to everything. They’ve decided, you know, they’ve come up with their decision, so, you believe it as well.”)

*Offense Characteristics* heavily influenced the participant’s *Initial Reaction* to the accusations, the third category to emerge in this period. That is, participants’ reactions were more intense and more negative when reacting to *Offense Types* and *Victim Characteristics* that they perceived as more severe (e.g., contact offenses, child victim). Additionally, if there had been *Consequences* to the accusations, and especially when the Criminal Justice System was involved, participants usually displayed a negative, rather than indifferent, *Initial Reaction.* Such an *Initial Reaction* could be behavioral (e.g., physically distancing oneself), affective (e.g., anger, sadness, disgust), and cognitive (e.g., confusion, worsened view of one’s partner). How a participant reacted to the accusations was often in part determined by their *Partner’s Reaction*. Some participants reported believing their partner when they denied the accusations, and thus did not react strongly. A confession, on the other hand mostly led to the participant believing the accusations. Overall, participants reported their *Initial Reaction* to be either negative (*n* = 14), indifferent (8) or positive (*n* = 1). A predominantly negative *Initial Reaction* usually eventually led the participant to believe the accusations.

The next category to emerge was *Telling Others*. Many participants (*n* = 10) reported that a negative *Initial Reaction* of shame, guilt, and a fear of judgement caused them to hide the accusations from others (see arrow on the left-hand side of the model). This led to a lack of social support outside of the participant’s relationship with their partner. When telling others about the accusations, participants confided in either their friends (*n* = 9), family (*n* = 6), or professionals (*n* = 6), for example, a therapist or law enforcement personnel. Some participants confided in multiple groups or individuals. For those who confided in others about the accusations, *Others’ Reaction* played an important role in their belief in the accusations with participants tending to mirror others’ reactions (i.e., *n* = 5 mirrored disbelief and *n* = 10 took the accusations seriously). In some cases, it also influenced the belief of those who tried to hide the accusations from others, if the accusations were revealed against their will (*n* = 3; e.g., through vigilantes or media exposure), as depicted by the arrow titled “*Accusations are revealed*”.

Critically, each category in this period mentioned above has a significant impact on the participant’s *Belief in the Accusations*. Those participants who exhibited *Belief* in the accusations (*n* = 13) described their partner’s confession (as described in *Partner’s Reaction*) to be the most important factor in their unequivocal *Belief* in the accusations. Some also reported favorable *Victim Characteristics* (e.g., trust in the accuser due to emotionality), as mentioned earlier in this period, and seeing evidence to have led them to believe the accusations. On the other hand, those who reported a *Disbelief* in the accusations (*n* = 10) supported and justified this by discrediting either the accusations themselves (*n* = 3; e.g., because they are unrealistic, not plausible etc.), the accuser or source (*n* = 3; e.g., because of unfavorable *Victim Characteristics*) or both (*n* = 4).

### Period 4: Relationship Decision-Making (see Figure 4)

**Figure 4.**
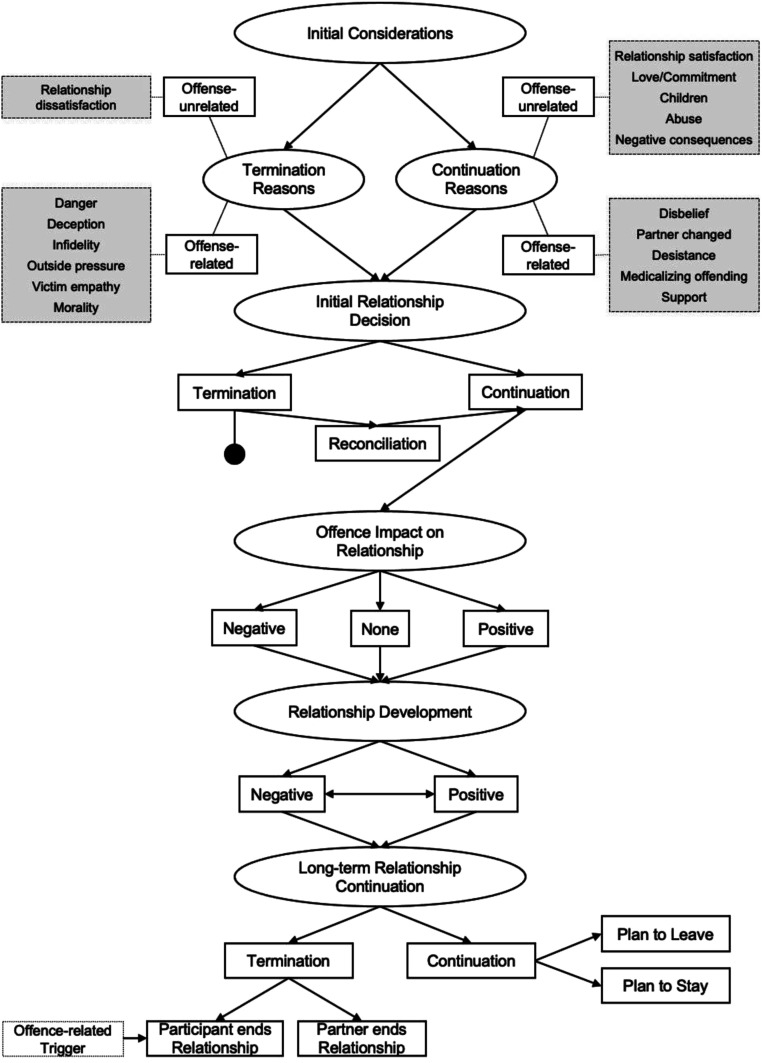
Period 4: Relationship decision-making.

The data which emerged regarding the participants’ decision to stay with or leave the relationship was divided into six main categories, with additional subordinate concepts (see Figure 4).

The first category to emerge was the *Initial Consideration* participants reported when considering *Termination Reasons* and *Continuation Reasons*. *Termination Reasons* (see left-hand side of the model) considered by participants, following the knowledge of accusations, were either *offense-related* or *-unrelated*. The only *offense-unrelated* reason for relationship termination considered by participants was relationship dissatisfaction, often arising from low *Relationship Quality* preceding the accusations (see Period 2; e.g., “I was already dissatisfied with certain aspects of the relationship”). However, most *Termination Reasons* considered by participants were directly related to the alleged offense, and participants only contemplated these if they had established a *Belief* in the accusations (see Period 3). Participants described considering leaving the relationship because of perceived *Danger*. Here, they stated viewing their partner as a dangerous person, and wanting to protect themselves and possibly their children (e.g., “I know what you’re doing to other people’s children. I don’t trust you around my own”). Participants judged their partner to be more dangerous towards themselves if they had been accused of a contact offense against an adult, and more dangerous towards their children if the participant had been accused of any offense a child, particularly if the accusations pertained a contact offense. Participants also reported the *Deception* associated with their partner’s alleged offending behavior to be a reason why they considered terminating the relationship. This was especially the case when their partner hid the offenses over a long period of time or denied accusations when the participant believed these to be true (e.g., “Our whole life felt like a lie”), leading participants to re-evaluate the relationship. Another reason for relationship dissolution considered by participants was perceived *Infidelity* because of the sexual nature of the alleged offense. Participants described feeling as if their partner had cheated on them, or that their partner offended because of a lack of attraction towards them (e.g., “She basically cheated on me”). The accusations against participants’ partners were only viewed as *Infidelity* if the victim was an adult (i.e., not a child) and in cases where the alleged offense took place while the participant was in the relationship with their partner. Additionally, some participants experienced *Outside pressure* to end the relationship, from friends, family, or their community (see *Others’ Reaction* in Period 3) or from intervening professionals. This was exacerbated in cases where the participant’s partner was arrested or convicted (see *CJS involvement* in Period 3), which usually meant that a greater proportion of the community was aware of the offense or accusations or gave them more credibility. Interaction with intervening professionals was often stated to be dismissive and even hostile (e.g., “The whole system is very, very anti-women, anti-children, in fact”). *Outside pressure* was especially salient where social services were involved to safeguard the participants’ children (e.g., “So they just gotta focus on making sure that the women run as fast as they can and take the children with them”), or in cases where the media reported on the accusations. Participants also reported considering relationship termination because of a sense of empathy, solidarity, and even guilt towards the victim (e.g., “I was just somehow feeling guilty. Like, I felt like I had to make it up to some way”). The last offense-related reason participants considered for terminating their relationship was *Morality*, a sense that leaving their partner after finding out about the accusations was the ‘right thing to do’ (e.g., “I don’t think I thought I had a choice”).

*Continuation Reasons* (see right-hand side of the model) considered by participants were similarly either *offense-unrelated* or -*related*. Participants mentioned a greater variety of *offense-unrelated* reasons for considering relationship continuation versus termination. *Relationship satisfaction*, arising from high *Relationship Quality* (see Period 2) was a main offense-unrelated reason for considering relationship continuation (e.g., “I was very, very happy with him, and at this point I can say I’m still happy to be with him”). Participants also stated the *Love* or *Commitment* they felt towards their partner to be a reason for considering relationship continuation. This was usually the case when the participant had been in the relationship for a long time, and especially when the participant was married (e.g., “When I said my wedding vows, I meant them”). For participants in such long-term committed relationships, having *Children* with their partner was also a commonly named reason for considering relationship continuation (e.g., “I’ve remained supportive of him for the sake of the children. But I don’t think I could forgive it, it’s just too horrible”). Additionally, some participants reported the *Abuse* they were subjected to from their partner (see Period 2), to be an important reason for considering continuing the relationship. Here, participants described being manipulated into thinking no one else would love them or being isolated by their partner and thus lacking a support system (e.g., “He would constantly say things like, ‘No one’s gonna love you like I do’”). The last *offense-unrelated* reason for consideration of relationship continuation, as stated by our participants, were *Negative consequences* of leaving the relationship, such as their partner retaliating or negative cultural implications of having been in a “failed” relationship.

Participants also named several *offense-related* relationship *Continuation Reasons.* The main *offense-related* reason participants described was a *Disbelief* or discrediting of the accusations, as outlined in-depth in Period 3. If participants disbelieved or discredited the accusations, they were likely to report not seeing a reason to consider ending the relationship at all. However, some participants also remained in the relationship despite believing the accusations to be true. For example, some of these participants believed that their *partner had changed* and cited this to be a reason they considered staying in the relationship. This was especially the case when the alleged offense had occurred before they entered the relationship. The more time had passed since the accusations the more likely participants were to judge that this did not reflect who their partner was when they were in the relationship, especially if their partner had actively worked on themselves, for example by attending therapy (e.g., “He did a lot of things to change himself. So, how can I judge him now?”). Additionally, participants reported wanting to aid their partner’s *Desistance* as a reason for considering relationship continuation. Here, participants emphasized the importance of relationships in preventing future offending behavior (e.g., “I just think that women who stay need to be given a bit of respect and credit. And also, when you look at the figures of reduction in reoffending and rehabilitation, that’s what’s needed”). Such a focus on *Desistance* was also often tied to a *Medicalization* of the offense. For instance, those whose partners were accused of committing image-based internet offenses against children commonly viewed this behavior as arising from an escalating pornography addiction (e.g., “Any other kind of addiction or issue, the person is treated, except this”). Lastly, participants described the *Support* they received to be a reason for considering relationship continuation. Usually, *Support* came from friends, family, or self-organized peer support groups, as professional support (e.g., therapy) was not always accessible. However, in contrast to the *Outside pressure* from intervening professionals some participants noted as a reason for relationship termination, here, some participants stated that intervening professionals were helpful and provided *Support*. For instance, participants reported being reassured that staying with their partner was an acceptable choice and that other people also stay with their partner following an alleged offense (e.g., “And she said to me, ‘Well, about 50% of couples make it through this.’”).

Overall, all participants named more than one reason for considering either staying with or leaving their partner. Thus, the *Initial Relationship Decision* was usually made after weighing up *Termination Reasons* and *Continuation Reasons*, often both *offense-related* and *-unrelated*. Eventually, seven participants decided for immediate relationship *Termination* (see left-hand side of the model). Some of these participants (*n* = 4) terminated the relationship initially after hearing about the accusations, but later decided to reconcile with their partner as marked by the *Reconciliation* box. Often, participants reported *Reconciliation* to be the result of being charmed or manipulated into re-entering the relationship by their partner. The remaining participants (*n* = 16) initially decided to engage in a *Continuation* of the relationship (see right-hand side of the model).

For those participants who continued the relationship, there was often a lasting *Offense Impact on the Relationship*. Participants usually reported the offense as having had a negative impact (*n* = 14; e.g., conflict, stress, not being able to forgive offense) on the relationship, with only one participant describing a positive impact (increased trust and intensity). For some participants (*n* = 5), the offense did not have any impact on their relationship. This was usually the case when there were other issues in the relationship, such as abuse, which seemed more pressing.

The impact of the offense on the relationship usually also influenced further *Relationship Development*. This was either predominantly negative (*n* = 13; e.g., escalating abuse, increased conflict, dissatisfaction) or positive (*n* = 5; e.g., rebuilding relationship, regaining trust). One participant reported that the *Relationship Development* was initially positive but later turned negative, as depicted by the arrow connecting the two pathways.

A positive *Relationship Development* made *Long-term Relationship Continuation* more likely, while a negative *Relationship Development* made it less likely. *Long-term Relationship Continuation,* the final category to emerge in this period, described the participants’ relationship status with their partner at the time of interview. Most participants (*n* = 14) did not continue the relationship long-term, and were not in the relationship anymore (i.e., *Termination*), at the time of interview. In a few cases (*n* = 3), the participant’s partner ended the relationship due to reasons unrelated to the offense. The participants who ended the relationship with their partner after initially continuing it earlier on in this period (*n* = 11), reported escalating abuse, a lack of trust arising from the offense, and relationship dissatisfaction as the main reasons for their decision to leave the relationship. For those who terminated the relationship, this was also sometimes influenced by an *Offense-Related Trigger* (*n* = 4). Such triggers were information that the participant received about the alleged offense after initially finding out about it which subsequently changed their perception of the offense (e.g., finding evidence, having contact with the victim). Very few of the participants (*n* = 6) continued the relationship long-term and were still in the relationship at the time of interview. These participants either planned to stay in the relationship (*n* = 5), usually because of high relationship satisfaction, or were planning to leave the relationship (*n* = 1), because they were still affected by the accusations but not ready to leave their partner yet.

### Pathways Followed by Participants

Each participant’s progression through the model was plotted to track potential discrete routes or *pathways* followed by non-offending partners of individuals who are accused of sexual offending in their relationship decision-making. However, the sample was too heterogenous for us to identify any distinct patterns.

## Discussion

Using interviews with current and ex-partners of individuals who have been accused of sexual offending, we developed the first descriptive model of relationship decision-making in non-offending partners. Our model’s key strength lies in its detailed account of why non-offending partners stayed in or left their relationship after finding out that their partner had been accused of a sexual offense. It clearly documents how affective, behavioral, cognitive, and situational factors that arose before, during, and after finding out about the accusations, contributed to such decision-making in our sample. Here, it is sufficiently sensitive to account for the diversity of non-offending partners. In the following section, we interpret the most important aspects of our model before discussing potential practical implications. Finally, we consider its key strengths and limitations. Directions for future research are outlined throughout.

First, our research suggests that relationship decision-making in non-offending partners is dependent on a large variety of factors, not all of which are directly related to the alleged offense, but rather connected to the participant’s life experiences prior to and during the relationship. This is especially the case for participants whose partners did not have any interaction with Criminal Justice System-related agencies, for instance those whose partners were accused but never arrested or convicted, and who subsequently may rely on their relationship experience rather than on outside factors. An especially notable reason for staying with their partner, as reported by our participants, which is unrelated to the accusations, was high relationship quality and arising *Relationship satisfaction*. It has been firmly established that such satisfaction, alongside other factors stated by our participants (e.g., *Commitment*, having *Children* with their partner, fear of *Negative consequences* of relationship dissolution), is one of the most common reasons for relationship continuation in the general population (Joel et al., 2018; Le et al., 2010). Similarly, the main offense-unrelated reason for leaving the relationship endorsed by our sample, *Relationship dissatisfaction*, has been found to also be a primary reason for relationship dissolution in the general population (Amato & Previti, 2003; Machia & Ogolsky, 2021). Most saliently, however, our model clearly demonstrates that participants were influenced in their decision-making by factors unique to their situation as non-offending partners. In particular, it accounts for the wide range of factors associated with the accusations themselves (e.g., perceived *Danger, victim empathy*), as well as how these affected the reaction to the accusations by others (e.g., *Outside pressure* to end the relationship, or *Support* to continue it) and how the sum of these factors collectively influenced the participant’s assessment of their situation. Here, whether the participant believed the accusations to be true or discredited them appeared to be the most notable factor shaping their decision-making process. Perhaps surprisingly, despite its impact on such a *Belief* in the accusations, no pattern emerged to distinguish those whose partners were “only” accused of offending from those whose partners were formally investigated or even convicted. Future research may be conducted to differentiate between these two populations to further understand possible differences and effectively address their individual needs.

The development of our model has several practical implications for practitioners working with non-offending partners and ex-partners, as well as those working with individuals who have sexually offended who are in relationships. First, while some organizations offer services to families of those who have offended (e.g., The Lucy Faithfull Foundation, *n*. d.) there are currently no targeted support resources for non-offending partners in England and Wales. Non-offending partners in our sample and in previous research (Duncan et al., 2022) reported facing various barriers (often financial) to accessing the limited support available and thus relying on self-organized peer support groups. This is concerning given the uniqueness and complexity of their situation demonstrated by our model and the psychological distress they experience as a result of their partner’s offending or accusations against their partner (Duncan et al., 2020). Our model may be used to inform potential support programs for both those whose partners have been accused or convicted. For example, such programs could use our model to help non-offending partners understand the variety of factors which may be related to their stay/leave decision and make the best possible decision for themselves and their families. Similarly, our model may also be of use to clinicians (e.g., counsellors) who may be approached by clients with similar experiences to those outlined in this paper. For these practitioners, especially those who have no experience in working with non-offending partners, our research could provide a first overview of their client’s unique situation and the complexity of the relationship decision-making process. Thus, it may assist clinicians in providing the best possible care for non-offending partners.

A second implication arises for Criminal Justice System-related organizations. Non-offending partners often encounter multiple agencies (e.g., police, child protection services) in the aftermath of their partner’s offense, and named their support, or lack thereof, to be crucial in relationship decision-making (see *Outside pressure* and *Support* in Period 3). Our participants reported interactions with these agencies to be inconsistent, ranging from supportive and empathetic to dismissive and hostile. Such particularly negative responses may cause or increase courtesy stigma experienced by non-offending partners, which is often exacerbated by Criminal Justice System-related agencies (Duncan et al., 2020; Liddell & Taylor, 2015). However, there is currently no consistent guidance available to Criminal Justice System related agencies on how to approach non-offending partners. Our model may inform codes of practice – like the Code of Practice for Victims of Crime in England and Wales (Ministry of Justice, 2020) – or training programs for professionals within these organizations. These may demonstrate the complexity of the relationship decision-making process and the diversity of the population and could enhance perspective-taking towards non-offending partners and thus reduce the courtesy stigma they experience.

In cases where someone’s partner was convicted of a sexual offense, rather than merely accused, a final practical implication of our model may be its potential impact on rehabilitation frameworks, such as the Risk-Need-Responsivity Model (Bonta & Andrews, 2017) and the Good Lives Model (Ward & Gannon, 2006), which commonly focus on relationships. Due to the demonstrated efficacy of treatment programs based upon such frameworks (e.g., Hanson et al., 2009) they are widely used, for example in the US (e.g., Washington State Department of Corrections, 2022) and the UK (H.M. Prison and Probation Service, 2021). Here, our model could illustrate to the treatment provider and the individual who has offended the complexity and difficulty of the decision the non-offending partner had to make, to promote greater empathy and subsequently improve relationship quality. Should a non-offending partner decide to stay in the relationship, treatment programs may also consider a greater involvement of the non-offending partner, in cases in which this is in both partners’ best interest. This could be achieved, for instance, by incorporating couples treatment to strengthen the relationship which, as shown in our model, may be particularly susceptible to breakdown after accusations or an offense have been disclosed. Such couples treatment may also address intimacy deficits which are commonly linked to offending behaviour (for a review see Martin & Tardif, 2014). Additionally, as the accusations against seven participants’ partners in our sample pertained offenses which were said to have taken place prior to the relationship, our model may also give insight to those who have offended about how to broach the topic of having committed an offense to a prospective or new partner. For instance, treatment providers may consider discussing with the person who has offended the importance of the source from whom the non-offending partner hears about the accusations or preparing them for potential outcomes as applicable to the specific circumstances surrounding their relationship and the offense.

The primary strength of our research is its novelty as it presents, to our knowledge, the first model of relationship stay/leave decision-making in non-offending partners of individuals accused of sexual offending. As such, it gives a detailed description of affective, behavioral, cognitive, and contextual factors contributing to why our participants decided to stay with or leave their partner after finding out about the accusations against them. Here, it is flexible enough to accommodate the diversity of our sample.

Despite this strength and the potential practical utility of the model there are, however, some limitations that require discussion. First, although our model is sufficiently saturated to account for the decision-making process of each participant in our sample, we were unable to find distinct *pathways* through the model followed by different “types” of non-offending partners. A potential explanation for this may be the diversity of non-offending partners as a group, when compared to the samples methodologically similar research has examined (e.g., Collins et al., 2022; Ford et al., 2021). For example, offense chain models developed using Grounded Theory typically include individuals convicted of and detained for a certain offense (e.g., Gannon et al., 2008; Tyler et al., 2014) whereas we included anyone whose partner or ex-partner was accused of any sexual offense. A model of relationship decision-making must be very sensitive to account for such heterogeneity. While our sample size was not unusually small for Grounded Theory research (Charmaz, 2014), and we sought to include non-offending partners with a range of different experiences, it is likely that certain groups were underrepresented which may explain why we were unable to identify pathways through the model. For instance, only one participants’ partner was convicted of committing contact sexual offenses against children, and some relatively common offenses (e.g., exposure, voyeurism) were not represented at all. Our sample did also not include any cases of intrafamilial abuse. This contrasts with the overall literature on non-offending partners which has historically disproportionately focused on, and blamed, non-offending mothers (Azzopardi et al., 2018). Nevertheless, as prior research has established (e.g., Gannon et al., 2008; Polaschek et al., 2001), a key strength of Grounded Theory methodology is its ability for future modification of models in response to additional data (Strauss & Corbin, 1990). Thus, further research may be conducted with additional samples of non-offending partners and ex-partners of individuals who have sexually offended or been accused of sexual offending to validate and refine the model and any potential pathways.

In addition to the limited sample described above, there are several limitations inherent in Grounded Theory which may have impacted the validity and reliability of our model. Most important is our reliance on retrospective self-reports given by non-offending partners. These may have been affected by memory distortions, self-deception, and impression management strategies. Crucially, most participants in our sample were not in the relationship anymore at the time of interview. This could have led to a distorted view of the situation, such as a negative re-definition of the relationship (e.g., arising from arguments or jealousy) commonly experienced after a breakup (Kellas et al., 2008). Future research may wish to focus on a balanced sample of those non-offending partners who are currently still in the relationship and those who are not to control for such effects. Finally, our model may represent some researcher bias, for instance, reflecting our pre-existing hypotheses and knowledge from the wider literature on non-offending partners or sexual offending. Given the dearth of existing research and paired with the principles of Grounded Theory employed to reduce such bias (i.e., simultaneous inductive and deductive analysis; reflexivity), we believe these strategies sufficiently minimized researcher biases. Nonetheless, future research should be completed to cross-validate the model and examine pathways to relationship stay/leave decision-making in non-offending partners of those who have sexually offended.

In conclusion, our model represents, to our knowledge, the first attempt to describe the relationship decision-making process in non-offending partners of individuals who have sexually offended. Given the dearth of research and theory examining the experiences of this population, using Grounded Theory methodology allowed us to develop a flexible yet detailed model based upon the individual narratives of non-offending partners. We hope that practitioners and researchers working with this population will build upon our research and use it to develop their understanding of non-offending partners’ experiences.

## References

[bibr1-10790632231159075] Amato P. R. Previti D. (2003). People’s reasons for divorcing: Gender, social class, the life course, and adjustment. Journal of Family Issues, 24(5), 602–626. 10.1177/0192513x03024005002

[bibr2-10790632231159075] Azzopardi C. Alaggia R. Fallon B. (2018). From Freud to feminism: Gendered constructions of blame across theories of child sexual abuse. Journal of Child Sexual Abuse, 27(3), 254–275. 10.1080/10538712.2017.1390717 29161221

[bibr3-10790632231159075] Bailey D. J. S. (2018). A life of grief: An exploration of disenfranchised grief in sex offender significant others. American Journal of Criminal Justice, 43(3), 641–667. 10.1007/s12103-017-9416-4

[bibr4-10790632231159075] Bonta J. Andrews D. A. (2017). The psychology of criminal conduct (6th ed.). Routledge.

[bibr5-10790632231159075] Brennan C. L. Swartout K. M. Cook S. L. Parrott D. J. (2018). A qualitative analysis of offenders’ emotional responses to perpetrating sexual assault. Sexual Abuse, 30(4), 393–412. 10.1177/1079063216667917 27591752

[bibr6-10790632231159075] Brown M. (2018). An exploration of the challenges families experience when a family member is convicted of sex offence [Master’s dissertation]. https://howardleague.org/wp-content/uploads/2018/12/An-exploration-of-the-challenges-families-experience-when-a-family-member-is-convicted-of-sex-offence.pdf

[bibr7-10790632231159075] Burton V. S. Jr. Cullen F. T. Travis L. F. III. (1987). The collateral consequences of a felony conviction: A national study of state statutes. Federal Probation, 51(3), 52–60.

[bibr8-10790632231159075] Cahalane H. Duff S. (2018). A qualitative analysis of nonoffending partners’ experiences and perceptions following a psychoeducational group intervention. Journal of Sexual AggressionAggression, 24(1), 66–79. 10.1080/13552600.2017.1384264

[bibr9-10790632231159075] Cassidy K. Kabbash L. Ronis S. T. (2021). A qualitative content analysis of an online support forum for family members of individuals with reported histories of sexual offences. The Canadian Journal of Human Sexuality, 30(2), 232–242. 10.3138/cjhs.2021-0012

[bibr10-10790632231159075] Charmaz K. (2014). Constructing grounded theory (2nd ed.). SAGE.

[bibr12-10790632231159075] Collins J. Barnoux M. Langdon P. E. (2022). A preliminary firesetting offence chain for adults with intellectual and other developmental disabilities. Journal of Intellectual and Developmental Disability. 10.3109/13668250.2022.2037186 39815912

[bibr15-10790632231159075] Crown Prosecution Service (2022). CPS data summary Quarter 4 2021 – 2022. https://www.cps.gov.uk/publication/cps-data-summary-quarter-4-2021-2022

[bibr200-10790632231159075] *Criminal Justice and Courts Act* *2015*, c. 2. https://www.legislation.gov.uk/ukpga/2015/2

[bibr16-10790632231159075] Duff S. Wakefield N. Croft A. Perry L. Valavanis S. Wright L. (2017). A service for non-offending partners of male sexual offenders. Journal of Forensic Practice, 19(4), 288–295. 10.1108/JFP-02-2017-0004

[bibr17-10790632231159075] Duncan K. Wakeham A. Winder B. Armitage R. Roberts L. Blagden N. (2020). The experiences of non-offending partners of individuals who have committed sexual offences: Recommendations for practitioners and stakeholders. University of Huddersfield. https://huddersfield.box.com/s/1sumdnyq9yjkgwhw0axzvgt7e2rfgcih

[bibr18-10790632231159075] Duncan K. Wakeham A. Winder B. Blagden N. Armitage R. (2022). “Grieving someone who’s still alive, that’s hard”: The experiences of non-offending partners of individuals who have sexually offended: An IPA study. Journal of Sexual Aggression, 28(3). 281–295. 10.1080/13552600.2021.2024611

[bibr19-10790632231159075] Evans D. Trahan A. Laird K. (2021). Shame and blame: Secondary stigma among families of convicted sex offenders. Criminology & Criminal Justice, 23(1). 78–97. 10.1177/17488958211017391

[bibr20-10790632231159075] Farkas M. A. Miller G. (2007). Reentry and reintegration: Challenges faced by the families of convicted sex offenders. Federal Sentencing Reporter, 20(2), 88–92. 10.1525/fsr.2007.20.2.88

[bibr21-10790632231159075] Farmer M. McAlinden A. M. Maruna S. (2015). Understanding desistance from sexual offending: A thematic review of research findings. Probation Journal, 62(4), 320–335. 10.1177/0264550515600545

[bibr22-10790632231159075] Ford J. Alleyne E. Blake E. Somers A. (2021). A descriptive model of the offence process for animal abusers: Evidence from a community sample. Psychology, Crime and Law Law, 27(4), 324–340. 10.1080/1068316X.2020.1798430

[bibr23-10790632231159075] Gannon T. A. Rose M. R. Ward T. (2008). A descriptive model of the offense process for female sexual offenders. Sexual Abuse: A Journal of Research and Treatment, 20(3), 352–374. 10.1177/1079063208322495 18775843

[bibr24-10790632231159075] Glaser B. G. Strauss A. L. (1967). The discovery of grounded theory: Strategies for qualitative research. AldineTransaction.

[bibr25-10790632231159075] Goffman E. (1963). Stigma: Notes on the management of spoiled identity. Prentice-Hall.

[bibr26-10790632231159075] Hanson R. K. Bourgon G. Helmus L. Hodgson S. (2009). The principles of effective correctional treatment also apply to sexual offenders. Criminal Justice and Behavior, 36(9), 865–891. 10.1177/0093854809338545

[bibr27-10790632231159075] Hanson R. K. Morton-Bourgon K. E. (2005). The characteristics of persistent sexual offenders: A meta-analysis of recidivism studies. Journal Of Consulting And Clinical Psychology, 73(6), 1154–1163. 10.1037/0022-006X.73.6.1154 16392988

[bibr28-10790632231159075] Iffland J. A. Berner W. Dekker A. Briken P. (2016). What keeps them together? Insights into sex offender couples using qualitative content analyses. Journal of Sex and Marital Therapy Marital Therapy, 42(6), 534–551. 10.1080/0092623X.2015.1079757 26280194

[bibr29-10790632231159075] Jeglic E. L. Spada A. Mercado C. C. (2013). An examination of suicide attempts among incarcerated sex offenders. Sexual Abuse: A Journal of Research and Treatment, 25(1), 21–40. 10.1177/1079063212447201 22661392

[bibr30-10790632231159075] Joel S. MacDonald G. Page-Gould E. (2018). Wanting to stay and wanting to go: Unpacking the content and structure of relationship stay/leave decision processes. Social Psychological and Personality Science, 9(6), 631–644. 10.1177/1948550617722834

[bibr31-10790632231159075] Jones C. Woodlock D. Salter M. (2021). Evaluation of PartnerSPEAK. https://uploadsssl.webflow.com/526b7ac56d0c50607100026b/61bc5530e2d03c459f856b57_PartnerSPEAKevaluation-Nomoreedits.pdf

[bibr32-10790632231159075] Jones E. Giles D. C. (2022). Women who remain in relationships with registered sexual offenders: Analysis of forum discussion. Journal of Community and Applied Social PsychologyPsychology, 32(1), 109–118. 10.1002/CASP.2558

[bibr33-10790632231159075] Kellas J. K. Bean D. Cunningham C. Cheng K. Y. (2008). The ex-files: Trajectories, turning points, and adjustment in the development of post-dissolutional relationships. Journal of Social and Personal Relationships, 25(1), 23–50. 10.1177/0265407507086804

[bibr34-10790632231159075] Kilmer A. Leon C. S. (2017). ‘Nobody worries about our children’: Unseen impacts of sex offender registration on families with school-age children and implications for desistance. A Critical Journal of Crime, Law and Society, 30(2), 181–201. 10.1080/1478601x.2017.1299852

[bibr35-10790632231159075] Larson M. Sweeten G. Piquero A. R. (2016). With or without you? Contextualizing the impact of romantic relationship breakup and crime among serious adolescent offenders. Journal of Youth and Adolescence, 45(1), 54–72. 10.1007/s10964-015-0318-9 26092231

[bibr36-10790632231159075] Le B. Dove N. L. Agnew C. R. Korn M. S. Mutso A. A. (2010). Predicting nonmarital romantic relationship dissolution: A meta-analytic synthesis. Personal Relationships, 17(3), 377–390. 10.1111/j.1475-6811.2010.01285.x

[bibr37-10790632231159075] Liddell M. Taylor C. (2015). PartnerSPEAK Research Report: Women’s experiences of learning about the involvement of a partner possessing child abuse material in Australia. https://uploads-ssl.webflow.com/526b7ac56d0c50607100026b/61bc5424cdb556143a8edfe8_PSResearchReport2015.pdf

[bibr38-10790632231159075] Lytle R. Bailey D. J. S. ten Bensel T. (2017). We fought tooth and toenail: Exploring the dynamics of romantic relationships among sex offenders who have desisted. Criminal Justice Studies, 30(2), 117–135. 10.1080/1478601X.2017.1299322

[bibr39-10790632231159075] Machia L. V. Ogolsky B. G. (2021). The reasons people think about staying and leaving their romantic relationships: A mixed-method analysis. Personality and Social Psychology Bulletinl Psychology Bulletin, 47(8), 1279–1293. 10.1177/0146167220966903 33150849

[bibr40-10790632231159075] Malinen S. Willis G. M. Johnston L. (2013). Might informative media reporting of sexual offending influence community members' attitudes towards sex offenders? Psychology, Crime and Law, 20(6), 535–552. 10.1080/1068316X.2013.793770

[bibr41-10790632231159075] Martin G. M. Tardif M. (2014). What we do and don't know about sex offenders' intimacy dispositions. Aggression and Violent Behavior, 19(4), 372–382. 10.1016/j.avb.2014.06.002

[bibr42-10790632231159075] McAlinden A. M. Farmer M. Maruna S. (2017). Desistance from sexual offending: Do the mainstream theories apply? Criminology and Criminal Justicel Justice, 17(3), 266–283. 10.1177/1748895816670201

[bibr43-10790632231159075] Ministry of Justice (2020). Code of practice for victims of crime in England and Wales. https://assets.publishing.service.gov.uk/government/uploads/system/uploads/attach ment_data/file/936239/victims-code-2020.pdf

[bibr44-10790632231159075] Olver M. E. Barlow A. A. (2010). Public attitudes toward sex offenders and their relationship to personality traits and demographic characteristics. Behavioral Sciences and the Law, 28(6), 832–849. 10.1002/bsl.959 20857417

[bibr45-10790632231159075] Polaschek D. L. L. Hudson S. M. Ward T. Siegert R. J. (2001). Rapists‘ offense processes: A preliminary descriptive model. Journal of Interpersonal Violence, 16(6), 523–544. 10.1177/088626001016006003

[bibr201-10790632231159075] *Protection of Children Act* *1978*, c. 37. https://www.legislation.gov.uk/ukpga/1978/37

[bibr46-10790632231159075] H.M Prison and Probation Service (2021). The HMPPS approach to the management and rehabilitation of people convicted of sexual offences. https://www.gov.uk/government/publications/the-hmpps-approach-to-the-management-and-rehabilitation-of-people-convicted-of-sexual-offences

[bibr48-10790632231159075] Quinn J. F. Forsyth C. J. Mullen-Quinn C. (2004). Societal reaction to sex offenders: A review of the origins and results of the myths surrounding their crimes and treatment amenability. Deviant Behavior, 25(3), 215–232. 10.1080/01639620490431147

[bibr50-10790632231159075] Rapp L. A. (2011). Women in romantic relationships with convicted sex offenders (Publication No. 3498536). [Doctoral dissertation, University of Delaware]. ProQuest Dissertations and Theses Global.

[bibr51-10790632231159075] Rogers D. L. Ferguson C. J. (2011). Punishment and rehabilitation attitudes toward sex offenders versus nonsexual offenders. Journal of Aggression, Maltreatment and Trauma, 20(4), 395–414. 10.1080/10926771.2011.570287

[bibr52-10790632231159075] Rothwell M. Fido D. Heym N. (2021). Perceptions around adult and child sex offenders and their rehabilitation as a function of education in forensic psychology independent of traditionalism and perpetrator sex. Forensic Science International: Mind And Law, 2(1). e100039, 10.1016/j.fsiml.2020.100039

[bibr53-10790632231159075] Schneider S. L. Wright R. C. (2004). Understanding denial in sexual offenders: A review of cognitive and motivational processes to avoid responsibility. Trauma, Violence, and Abuse, 5(1), 3–20. 10.1177/1524838003259320 15006294

[bibr60-10790632231159075] Shannon K. L. Pearce E. Swarbrick R. (2013). Factors influencing the development of an innovative service for women non-offending partners (NOPs) of male sexual offenders. Journal of Sexual Aggression, 19(3), 357–368. 10.1080/13552600.2012.729092

[bibr61-10790632231159075] Stinson J. D. Gonsalves V. (2014). Suicide attempts and self-harm behaviors in psychiatric sex offenders. Sexual Abuse: A Journal of Research and Treatment, 26(3), 252–270. 10.1177/1079063213486935 23657275

[bibr62-10790632231159075] Strauss A. Corbin J. (1998). Basics of qualitative research: Techniques and procedures for developing grounded theory (2nd ed.). SAGE.

[bibr63-10790632231159075] Strauss A. Corbin J. M. (1990). Basics of qualitative research: Grounded theory procedures and techniques. Sage Publications, Inc.

[bibr202-10790632231159075] *Sexual Offences Act* *2003*, c. 41. https://www.legislation.gov.uk/ukpga/2003/42

[bibr64-10790632231159075] Tewksbury R. (2005). Collateral consequences of sex offender registration. Journal of Contemporary Criminal Justice, 21(1), 67–81. 10.1177/1043986204271704

[bibr65-10790632231159075] Tewksbury R. Levenson J. (2009). Stress experiences of family members of registered sex offenders. Behavioral Sciences & the Law, 27(4), 611–626. 10.1002/bsl.878 19499594

[bibr66-10790632231159075] The Lucy Faithfull Foundation. (n.d.) . Inform – For families of people who have offended online. Rretrieved April 8. 2022, from. https://www.lucyfaithfull.org.uk/inform.htm

[bibr67-10790632231159075] Tyler N. Gannon T. A. Lockerbie L. King T. Dickens G. L. De Burca C. (2014). A firesetting offense chain for mentally disordered offenders. Criminal Justice and Behavior, 41(4), 512–530. 10.1177/0093854813510911

[bibr68-10790632231159075] U.S. Department of Justice, Office of Justice Programs, Bureau of Justice Statistics (2016). Female victims of sexual violence, 1994–2010. https://bjs.ojp.gov/content/pub/pdf/fvsv9410.pdf

[bibr69-10790632231159075] Wager N. M. Wager A. R. Wilson C. (2015). Circles South East’s programme for non- offending partners of child sex offenders: A preliminary outcome evaluation. Probation Journal, 62(4), 357–373. 10.1177/0264550515600541

[bibr70-10790632231159075] Ward T. Gannon T. A. (2006). Rehabilitation, etiology, and self-regulation: The comprehensive good lives model of treatment for sexual offenders. Aggression and Violent Behavior, 11(1), 77–94. 10.1016/j.avb.2005.06.001

[bibr71-10790632231159075] Ward T. Hudson S. M. Marshall W. L. (1995). Cognitive distortions and affective deficits in sex offenders: A cognitive deconstructionist interpretation. Sexual Abuse: A Journal of Research and Treatment, 7(1), 67–83. 10.1007/BF02254874

[bibr72-10790632231159075] Ward T. Polaschek D. L. L. Beech A. R. (2006). Theories of sexual offending. John Wiley & Sons, Ltd.

[bibr73-10790632231159075] Ware J. Blagden N. Harper C. (2020). Are categorical deniers different? Understanding demographic, personality, and psychological differences between denying and admitting individuals with sexual convictions. Deviant Behavior, 41(4), 399–412. 10.1080/01639625.2018.1558944

[bibr74-10790632231159075] Washington State Department of Corrections (2022). Sex offender treatment and assessment. https://www.doc.wa.gov/corrections/programs/sex-offender-treatment.htm#policies

[bibr75-10790632231159075] Willis G. M. Levenson J. S. Ward T. (2010). Desistance and attitudes towards sex offenders: Facilitation or hindrance? Journal of Family Violence, 25, 545–556. 10.1007/s10896-010-9314-8

